# Associations of Polygenic Risk Score for Late-Onset Alzheimer's Disease With Biomarkers

**DOI:** 10.3389/fnagi.2022.849443

**Published:** 2022-04-14

**Authors:** Qiaojun Li, Xingping Lv, Fei Jin, Kun Liao, Liyuan Gao, Jiayuan Xu

**Affiliations:** ^1^School of Information Engineering, Tianjin University of Commerce, Tianjin, China; ^2^School of Sciences, Tianjin University of Commerce, Tianjin, China; ^3^Department of Molecular Imaging, Qingdao Central Hospital, Qingdao University, Qingdao, China; ^4^Department of Radiology and Tianjin Key Laboratory of Functional Imaging, Tianjin Medical University General Hospital, Tianjin, China

**Keywords:** late onset Alzheimer's disease, polygenic risk score, biomarker, prediction, brain

## Abstract

Late-onset Alzheimer's disease (LOAD) is a common irreversible neurodegenerative disease with heterogeneous genetic characteristics. Identifying the biological biomarkers with the potential to predict the conversion from normal controls to LOAD is clinically important for early interventions of LOAD and clinical treatment. The polygenic risk score for LOAD (AD-PRS) has been reported the potential possibility for reliably identifying individuals with risk of developing LOAD recently. To investigate the external phenotype changes resulting from LOAD and the underlying etiology, we summarize the comprehensive associations of AD-PRS with multiple biomarkers, including neuroimaging, cerebrospinal fluid and plasma biomarkers, cardiovascular risk factors, cognitive behavior, and mental health. This systematic review helps improve the understanding of the biomarkers with potential predictive value for LOAD and further optimizing the prediction and accurate treatment of LOAD.

## Introduction

Alzheimer's disease (AD) which accounts for about 70% of dementia is an irreversible progressive polygenic neurodegenerative disease with insidious onset (Kametani and Hasegawa, 2018; Breijyeh and Karaman, 2020; Tank et al., 2022). By age at onset, AD can be classified into early-onset AD (EOAD) and late-onset AD (LOAD). EOAD is an autosomal dominant disease with heritability of more than 70% (Gatz et al., 2006; Wingo et al., 2012) and three responsible mutated genes, the amyloid protein precursor gene (*APP*), presenilin-1 gene (*PSEN1*), and presenilin-2 gene (*PSEN2*), were found to mainly dominate the production, aggregation, and clearance of amyloid β-protein (Aβ) (Cacace et al., 2016). Unlike the EOAD, LOAD occurs in more than 95% of the AD patients with a relatively complex polygenetic mechanism (Zhu et al., 2015; Xiao et al., 2017), and the related external phenotype changes in the very early stage. Although aducanumab can reduce the amyloid deposition in the brain and has been approved by Food and Drug Administration to treat Alzheimer's disease lately, however, controversy about it still exists (Selkoe, 2021; Servick, 2021). Therefore, identifying the biomarkers with the potential to predict the conversion from normal controls to LOAD and the progression of LOAD is clinically very important for early interventions.

In recent years, genome-wide association studies (GWAS) have been widely applied to study complex neuropsychiatric disorders (Ripke et al., 2014; Lello et al., 2019; van der Merwe et al., 2019; Levey et al., 2021; Peyrot and Price, 2021) and more than 200 susceptibility genetic variants have been identified to characterize the polygenetic architecture of LOAD (Chen et al., 2021). To overcome the small effect size of a single genetic variant, some polygenic methods have been developed to quantify the cumulative effects of multiple genetic variants related to complex diseases (Tan et al., 2018; Altmann et al., 2020; Choi et al., 2020), of which the polygenic risk score (PRS) is the most representative and widely used method (Wray et al., 2021). With the release of large-sample GWAS summary statistics for LOAD (Lambert et al., 2013; Weiner et al., 2015; Kunkle et al., 2019), AD-PRS, which measures the cumulative genome-wide-weighted effects of LOAD-risk genetic variants, is being increasingly used with multiple biomarkers to identify the underlying neurobiological mechanisms of LOAD.

**Figure 1:**
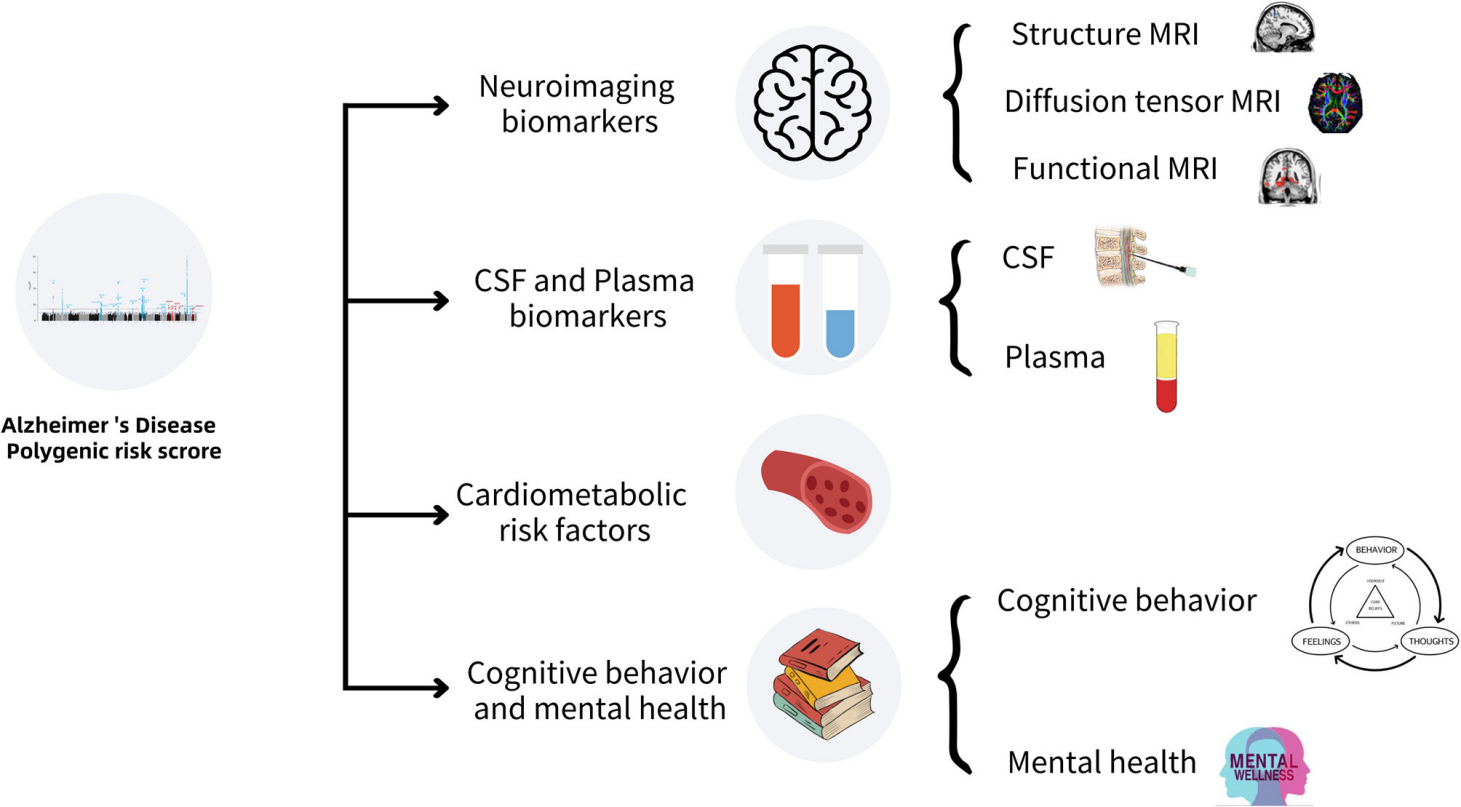
Association of AD-PRS with various biomarkers for LOAD.

In this review, we summarized the research progress of the associations of AD-PRS with multiple biomarkers, including neuroimaging, cerebrospinal fluid, and plasma, cardiovascular risk factors, cognitive behaviors, and mental health. This review is helpful to identify the biomarkers with the potential to predict the occurrence and development of LOAD, which is clinically important for the early diagnosis and interventions of this complex disease. A schematic summary of the related work in this review is shown in Figure 1 and Table 1.

**Table 1:** The work progress in the associations of AD-PRS with multiple biomarkers.

**Biomarker**	**Subfields**	**Variables**	**References**	**Program for PRS**	**Base sample**	**Target sample**	**Correlation**		**Regression**			**MRI coordinate**	
							**r/R2**	**Sig**.	**β/OR**	**Sig**.	**95%CI**	**Coordinate** **[x,y,z]**	**Sig**.
MRI	Structure MRI	GMV in hippocampus	Axelrud et al., 2018	PRSice	IGAP, *n =* 74,046	BHRC, *n =* 716			Left hippocampus: β = −0.301; right hippocampus: β = −0.319	-	Left hippocampus: [−0.434,−0.087]; right hippocampus: [−0.468,−0.072]		
		GMV in hippocampal subregions	Heidi et al., 2021	PRSice2	IGAP, *n =* 74,046	UKBB, *n =* 17,161			Left cornu ammonis: β = −0.0209; Right cornu ammonis: β = −0.0112	Left cornu ammonis: *p =* −0.000629; Right cornu ammonis: *p =* 0.068324			
		GMV in left precuneus	Li et al., 2018	PLINK	IGAP, *n =* 74,046	Recruited from society, *n =* 683						[−12, −51, 58.5]	*p* <0.05
		GMV in right cingulate gyrus	Li et al., 2018	PLINK	IGAP, *n =* 74,046	Recruited from society, *n =* 683						[6, 3, 33]	*p* <0.05
		GMV in right superior frontal gyrus	Li et al., 2018	PLINK	IGAP, *n =* 74,046	Recruited from society, *n =* 683						[6, 66, 1.5]	*p* <0.05
		GMV in right caudate	Li et al., 2018	PLINK	IGAP, *n =* 74,046	Recruited from society, *n =* 683						[−1.5 ,4.5, 1.5]	*p* <0.05
		CS in frontal pole	Xiao et al., 2017	PLINK	IGAP, *n =* 74,046	Recruited from society, *n =* 231			-	*p =* 0.029			
		CT in bilateral medial temporal cortex	Lee et al., 2021	PLINK	IGAP, *n =* 54,162	ADNI, *n =*217	-	-					
		CT in posterior cingulate cortex	Sabuncu et al., 2012	PLINK	ADNI, *n =* 745	ADNI, *n =* 204	*r =* −0.27	p <0.05					
		CT in entorhinal cortex	Harrison et al., 2016	-	IGAP, *n =* 74,046	UCLA Longevity Center, *n =* 45	unweighted risk score: *r =* −0.35; weighted risk score: *r =* −0.35	unweighted risk score: *p =* 0.009; weighted risk score: *p =* 0.009					
	Diffusion tensor MRI	FA in the right cingulum bundle	Foley et al., 2017	PLINK	IGAP, *n =* 54,162	CUBRIC, *n =* 272	R2 = 0.032	*p =* 0.009					
		FA and MD in inferior occipito-frontal fascicle	Harrison et al., 2020a	-	-	-	-	-					
		FA and MD in superior longitudinal fascicle	Harrison et al., 2020a	-	-	-	-	-					
		FA and MD in cingulum	Harrison et al., 2020a	-	-	-	-	-					
		FA and MD in corpus callosum	Harrison et al., 2020a	-	-	-	-	-					
		MNS of visual subnetwork	Mirza-Davies et al., 2021	PLINK	IGAP, *n =* 94,437	ALSPAC, *n =* 562	*r =* −0.19	*p =* 1.3E−5					
	Functional MRI	FC between precuneus and superior temporal gyrus	Axelrud et al., 2019	PRSice	IGAP, *n =* 74,046	BHRC, *n =* 636			discovery sample: β = 0.180; replication sample: β = 0.202	discovery sample: p-adjusted = 0.036; replication sample: *p =* 0.031			
		FC within temporal cortex	Su et al., 2017	gPLINK	-	Recruited from hospital, *n =* 218			left middle temporal gyrus: β = −0.3	left middle temporal gyrus p <0.001			
		Activation in episodic memory processing network	Zhan et al., 2016	-	-	ADNI, *n =* 68						[5, 8, 11]	
		Activation in hippocampus	Chandler et al., 2020	PLINK	CTGLAB, *n =* 455,258	YA-HCP, *n =* 608			β = 0.102	*p* = 0.016	[0.019, 0.186]		
		Activation in hippocampus ROI	Xiao et al., 2017	PLINK	IGAP, *n =* 74,046	Recruited from society, *n =* 231						Left hippocampal activition: [−39, −24, −15]; right hippocampal activition: [39, −21, 15]	Left hippocampal activition: p <0.05; right hippocampal activition: p <0.05
		CBF in frontal regions	Chandler et al., 2019	PLINK	IGAP, *n =* 74,046	Recruited from society, *n =*75			β = −0.232	*p =* 0.031			
			Chandler et al., 2021	PRSice	IGAP, *n =* 94,437	ADNI, *n =* 90			β = −0.38	*p =* 0.012	[−0.68, −0.09]		
		T-tau	Porter et al., 2018a	-	IGAP, *n =* 74,046	AIBL, *n =* 643	include APOE: *r =* 0.1949; exclude APOE: *r =* 0.1787	include APOE: *p =* 0.1499; exclude APOE: *p =* 0.0348					
		P-tau	Porter et al., 2018a	-	IGAP, *n =* 74,046	AIBL, *n =* 643	include APOE: *r =* 0.1543; exclude APOE: *r =* 0.2044	include APOE: *p =* 0.2563; exclude APOE: *p =* 0.0719					
		Ration of Aβ42/Aβ40	Li et al., 2020	PLINK	IGAP, *n =* 74,046	Recruited from hospital, *n =* 925	*r =* −0.25	p <0.001					
	Plasma	Clusterin	Morgan et al., 2017	-	IGAP, *n =* 74,046	Recruited from society, *n =* 93	PRS: *r =* 0.2; Immune specific PRS: *r =* 0.25	PRS: *p =* 0.05; Immune specific PRS: *p =* 0.02					
		Complement receptor 1 inhibitor	Morgan et al., 2017	-	IGAP, *n =* 74,046	Recruited from society, *n =* 93	Immune specific PRS: *r =* 0.22	Immune specific PRS: *p =* 0.05					
		C-reactive protein	Morgan et al., 2017	-	IGAP, *n =* 74,046	Recruited from society, *n =* 93	Immune specific PRS: *r =* 0.16	Immune specific PRS: *p =* 0.13					
		Osteopontin	Zhou et al., 2020	R	-	Recruited from hospital, *n =* 829			β = 0.673	*p =* 5.95E−04			
		Neurocan core protein	Zhou et al., 2020	R	-	Recruited from hospital, *n =* 829			β = 0.411	*p =* 1.94E−03			
		P-tau 181	Zettergren et al., 2021	-	IGAP, *n =* 962	ADNI, *n =* 818			include APOE: β = 0.18 ~ 0.19 exclude APOE: β = 0.05 ~ 0.11	include APOE: *p =* 3E−18 ~ 7E−15 exclude APOE: *p =* 3E−4 ~ 0.03			
		Diabetes	Richardson et al., 2019	-	-	UBKK , *n =* 334,398	-	-					
		Diastolic blood pressure	Richardson et al., 2019	-	-	UBKK , *n =* 334,398	-	-					
		Mid-life hypertension and obesity	Baumgart et al., 2015	-	-	-	-	-					
		Traumatic brain injury	Baumgart et al., 2015	-	-	-	-	-					
		Coronary heart disease	Elman et al., 2019	PLINK	IGAP, *n =* 74,046	VETSA, *n =* 1,329	-	-					
		PRS of Coronary artery disease	Elman et al., 2019	PLINK	IGAP, *n =* 74,046	VETSA, *n =* 1,329			O*R =* 1.38	*p =* 0.023	[1.05, 1.83]		
		Height and weight	Korologou-Linden et al., 2019b	PLINK	IGAP, *n =* 74,046	ALSPAC, *n =* 7,977			height-adjusted fat mass: β = 0.59% ; height-adjusted lean mass: β = 0.04 kg		height-adjusted fat mass: [−0.92, 2.11]; height-adjusted lean mass: [−0.03, 0.11]		
		Triglyceride	Korologou-Linden et al., 2019b	PLINK	IGAP, *n =* 74,046	ALSPAC, *n =* 7,977	-	-					
		Insulin and C-reactive protein	Korologou-Linden et al., 2019b	PLINK	IGAP, *n =* 74,046	ALSPAC, *n =* 7,977	-	-					
Cognitive behavior and mental health	Cognitive behavior	Immediate memory	Marden et al., 2016	-	IGAP, *n =* 74,046	HRS, *n =* 8,253			non-Hispanic whites: β = −0.058; non-Hispanic blacks: β = −0.050		non-Hispanic whites: [−0.074,−0.043]; non-Hispanic blacks: [−0.106,0.006]		
		Verbal episodic memory	Porter et al., 2018b	R	-	AIBL, *n =* 226	include APOE: *r =* −0.259; exclude APOE: *r =* −0.208	include APOE: *p =* 0.00003; exclude APOE: *p =* 0.004					
		General episodic memory	Li et al., 2018	PLINK	IGAP, *n =* 74,046	Recruited from society, *n =* 683			Working memory 2-back: β = −0.068; Working memory 3-back: β = −0.061	Working memory 2-back: *p =* 0.196 ; Working memory 3-back: *p =* 0.249			
		Total intelligence quotients	Korologou-Linden et al., 2019a	PLINK	IGAP, *n =* 74,046	ALSPAC, *n =* 5,525			β = −0.04	*p =* 0.002	[−0.07, −0.02]		
		Verbal intelligence quotients	Korologou-Linden et al., 2019a	PLINK	IGAP, *n =* 74,046	ALSPAC, *n =* 5,525			β = −0.04	*p =* 0.003	[−0.07, −0.01]		
		Performance intelligence quotients	Korologou-Linden et al., 2019a	PLINK	IGAP, *n =* 74,046	ALSPAC, *n =* 5,525			β = −0.03	*p =* 0.012	[−0.06, −0.01]		
		Economic behaviors	Shin et al., 2019	-	IGAP, *n =* 74,046	HRS, *n =*2936			hands-on assets: β = −0.3558; hands-off assets: β = 0.1114	hands-on assets: p <0.001; hands-off assets: p > 0.05			
			Ajnakina et al., 2020	PRSice	IGAP, *n =* 74,046	ELSA, *n =*7039			intermediate wealth: β = −0.13; low wealth: β = −0.21	intermediate wealth: *p =* 0.03; low wealth: *p* <0.001	intermediate wealth: [−0.24, −0.01]; low wealth: [−0.30, −0.07]		
	Mental health	Delusions	Creese et al., 2019	PRSice	PGC, *n =* 150,034	ADNI, *n =* 3,111			β = 1.18	*p =* 0.001	[1.06, 1.3]		
		Schizophrenia	Creese et al., 2019	PRSice	PGC, *n =* 150,034	ADNI, *n =* 3,111			Psychosis wide: O*R =* 1.14; Psychosis narrow: O*R =* 1.16	Psychosis wide: *p =* 0.003; Psychosis narrow: *p =* 0.002	Psychosis wide: [1.05, 1.23]; Psychosis narrow: [1.06, 1.28]		
		Hallucinations	Kusters et al., 2020	PRSice	IGAP, *n =* 74,046	PEG, *n* = 281; PW, *n* = 118			O*R =* 1.37		[1.03, 1.83]		
			Creese et al., 2019	PRSice	PGC, *n =* 150,034	ADNI, *n =* 3,111	-	-					
		Neuroticism	Duberstein et al., 2011	-	-	GEM, *n =* 767			O*R =* 1.36		[1.08, 1.71]		
			Terracciano and Sutin, 2019	-	-	-	-	-					
		Major depression disorder	Xu et al., 2018	PRSice	PGC *n =* 150,034; IGAP *n =* 74,046	ADNI, *n =* 322	-	-					

*ADNI, Alzheimer's Disease Neuroimaging Initiative; AIBL, Australian Imaging, Biomarkers and Lifestyle study; ALSPAC, Avon Longitudinal Study of Parents and Children; Aβ, amyloid β-protein; BHRC, Brazilian High Risk Study for Psychiatric Disorders; CBF, cerebral blood flow; CS, cortical surface; CSF, cerebrospinal fluid; CT, cortical thickness; CTGLAB, Complex Ttait Genetics Lab; CUBRIC, Cardiff University; Brain Research Imaging Centre; dMRI, diffusion tensor MRI; ELSA, English Longitudinal Study of Aging; FA, fractional anisotropy; FC, functional connectivity; GEM, Details of the Ginkgo Evaluation of Memory study; GMV, gray matter volume; H70, Gothenburg H70 Birth Cohort Studies; HRS, Health and Retirement Study; IGAP, International Genomics of Alzheimer's Project; MD, mean diffusivity; MNS, mean nodal strength; PEG, The Parkinson's Environment and Gene study; PGC, Psychiatric Genomics Consortium; PRS, polygenic risk score; P-tau, phosphorylated tau; PW, Norwegian ParkWest study; ROI, region of interest; sMRI, structure MRI; T-tau, total tau; UBKK, UK Biobank; UCLA Longevity Center, University of California of Los Angeles Longevity Center; VETSA, Vietnam Era Twin Study of Aging; YA-HCP, Young Human Connectome Project; - , indicates that the information is not mentioned in the original text*.

## Associations of AD-PRS With Neuroimaging Biomarkers

Exploring the structural and functional changes through medical imaging techniques is crucial for understanding LOAD development. Because of the advantages of safety and information abundance, magnetic resonance imaging (MRI) has become prominent among various medical imaging techniques. Of the various modalities of MRI, structure MRI (sMRI), diffusion tensor MRI (dMRI), and functional MRI (fMRI) have been mostly applied to study the underlying neural mechanism of LOAD and its clinical diagnosis and treatment by exploring the correlation between AD-PRS and brain phenotypes.

sMRI is one of the most important avenues to illustrate the brain morphological measures, for example, gray matter volume, cortical surface area, and cortical thickness. Studies have found that AD-PRS was associated with reduced gray matter volume (GMV) in the hippocampus (Axelrud et al., 2018) and its subregions (Heidi et al., 2021), left precuneus and right cingulate gyrus cortex (Li et al., 2018), whereas with increased GMV in the right superior frontal gyrus and caudate (Li et al., 2018). Meanwhile, AD-PRS was found to be associated with decreased surface area in the frontal pole (Xiao et al., 2017), decreased cortical thickness in the bilateral medial temporal cortices (Lee et al., 2021), posterior cingulate cortices (Sabuncu et al., 2012), and bilateral entorhinal cortices (Harrison et al., 2016). The changes of these brain regions are some of the most prominent early pathological features of LOAD and can be used as reliable predictive measures for the conversion from normal controls or mild-cognitive impairment to LOAD (Yang et al., 2012).

dMRI is mainly used to measure the microstructural integrity of the white matter through modeling-water diffusivity in the tissue microstructure (Kilimann et al., 2013), with fractional anisotropy (FA) and mean diffusivity as the two most used indices. AD-PRS is associated with decreased FA in the right cingulum bundle in healthy adults (Foley et al., 2017). AD-PRS was also found to be associated with reduced FA and increased mean diffusivity across the whole brain white matter tracts, notably in the inferior occipitofrontal fascicle, superior longitudinal fascicle, cingulum and corpus callosum in the AD patients (Harrison et al., 2020a). Recently, Mirza-Davies et al. (2021) found the visual subnetwork constructed based on dMRI was also correlated with AD-PRS.

fMRI was used to evaluate brain activity by detecting changes associated with blood flow (Smitha et al., 2017), referred to as the blood-oxygen-level-dependent (BOLD) signal in the brain-resting or task-based state. AD-PRS was found to be associated with increased functional connectivity between the right precuneus and the right superior temporal gyrus in the youths, which might impact memory performance and inhibitory control in early life (Axelrud et al., 2019). AD-PRS was also found to be associated with decreased functional connectivity within the temporal cortex in mild-cognitive impairment patients (Su et al., 2017). The hippocampal activation, mostly responsible for episodic memory processing, was severely impaired in the LOAD patients (Zhan et al., 2016; Xiao et al., 2017). However, contrary research findings have been reported between the AD-PRS and hippocampal activation. Chandler et al. (2020) found a significantly positive correlation and Xiao et al. (2017) found a significantly negative correlation during the episodic memory. This divergence may be due to the different task codings and sample size of the studies.

Arterial spin labeling was a functional MRI technology for measuring tissue perfusion to quantify the cerebral blood flow (CBF) in a given period with high time resolution (Rostami et al., 2014). There is a hypothesis proposing that insufficient CBF increases the risk of developing LOAD, leads to the decline of consciousness and dysfunction of LOAD, and even can be treated as an early antecedent of LOAD (Chandler et al., 2021). AD-PRS was found to be negatively correlated with CBF on many brain regions across the younger and older participants, including the frontal pole, middle frontal gyrus, inferior frontal gyrus, insular, frontal medial cortex, and orbitofrontal cortex (Chandler et al., 2019, 2021). These studies may shed light on exploring the key molecular processes that underpin LOAD.

All of the above findings together revealed the close relationship between the cumulative genetic risk of LOAD and the changes in the brain structure and function, providing new perspectives to explain the pathophysiology of LOAD. The combination of the neuroimaging biomarkers with AD-PRS to predict the LOAD development is attracting attention (Harrison et al., 2016, 2020b; Williams et al., 2021) and this is thought to be a promising step toward improving the very early identification of LOAD (Williams et al., 2021).

## Associations of AD-PRS With Cerebrospinal Fluid and Plasma Biomarkers

The concentration determination of Aβ, total tau (T-tau), and phosphorylated tau (P-tau) in the cerebrospinal fluid (CSF) are three classical biomarkers for the clinical diagnosis of LOAD (Lee et al., 2019; Shen et al., 2021). The changes of these measures in the brain occur more than 15 years before the onset of symptoms in LOAD patients (Bateman et al., 2012; Dementia, 2021). More studies devoted to the association analysis of AD-PRS and these biomarkers found that AD-PRS was not only correlated with the CSF levels of Aβ42, Aβ42/Aβ40, T-tau, and P-tau in the older adults (Porter et al., 2018a; Li et al., 2020), but could also predict the incidence rate of LOAD and the age at onset (Li et al., 2020). In addition, there was an interaction between AD-PRS and the Aβ42 pathology status to the neurofilament light (NfL) (Skoog et al., 2021). Moreover, the A/T/N criteria including a combined accumulation of amyloid plaques (A), neurofibrillary tangles composed of tau (T), and neurodegeneration (N) can predict the cognitive decline and clinical progression of LOAD (Soldan et al., 2019; Ebenau et al., 2020) and are recommended to be included in the diagnostic categories of LOAD (Foley et al., 2017). AD-PRS also showed a significant correlation with the A/T/N profiles (Ebenau et al., 2021). A study found that the integration of genetic risk across the AD biomarkers like A/T/N may improve the prediction of the disease progression (Moore et al., 2019).

Various inflammations occur in pathologically vulnerable brain regions in LOAD patients (Akiyama, 2000) and many plasma biomarkers of inflammation are useful for early diagnosis and monitoring the progression of LOAD (Kinney et al., 2018; Naveed et al., 2019). AD-PRS was found to be associated with various increased inflammatory biomarkers in the plasma, such as clusterin, complement receptor 1 inhibitor and C-reactive protein (Morgan et al., 2017), osteopontin and neurocan core protein (Zhou et al., 2020), and P-tau 181 (Zettergren et al., 2021). Similar to other biomarkers, the integration of AD-PRS and inflammatory biomarkers can also greatly improve the sensitivity and specificity of predicting LOAD. These findings not only facilitate the development of genetic tools for assessing the individual risk of LOAD but could also improve our understanding of the underlying mechanisms of this disease.

## Associations of AD-PRS With Cardiometabolic Risk Factors

Many cardiometabolic risk factors are implicated in the etiology of LOAD and are thought to lie on the pathways linking the genetic variants of LOAD (Korologou-Linden et al., 2019b). Of these factors, cardiovascular risk factors are found to increase the incidence of LOAD (Lin et al., 2019), which may be due to the high genetic association between LOAD and many cardiovascular diseases, such as hypertension (Baumgart et al., 2015), coronary heart disease (Elman et al., 2019), diabetes, and diastolic blood pressure (Richardson et al., 2019). AD-PRS was also found positively associated with other cardiometabolic risk factors such as traumatic brain injury, obesity, and hypertension in adults (Baumgart et al., 2015). However, these associations are not consistent throughout the whole life trajectory. For example, Korologou-linden et al. did not detect evidence to suggest that AD-PRS acts through childhood and adolescent cardiometabolic risk factors (Korologou-Linden et al., 2019b). More studies should be conducted in other large-birth cohorts to examine whether the genetic risk for Alzheimer's disease can be captured in early childhood. If not, further studies should examine whether and why these associations emerge only later, in adulthood, when the variation in the cardiometabolic risk factors is likely to be greater.

The combination of the genetic accumulation risk of LOAD and some vascular risk factors increased the predictive potential of LOAD for the shared genetic heritage (Li et al., 2016). The coronary artery disease (CAD) interacting with the LOAD pathology is highly heritable and CAD-PRS has been widely used to improve cardiovascular risk prediction (Wehby et al., 2018; Elliott et al., 2020; Levin and Rader, 2020). A healthy adult group with higher CAD-PRS and AD-PRS showed a significantly increased risk of developing amnestic mild-cognitive impairment (aMCI) (Elman et al., 2019), which is a state of cognitive deficit that is not severe enough to fulfill the criteria of dementia (Bennett et al., 2002) and showed a much higher probability of developing into LOAD (Chaudhury et al., 2019). In summary, AD-PRS, combined with the PRS of cardiovascular risk factors, has shown a superior predictive value of onset of aMCI and LOAD compared to the independent application of AD-PRS, indicating the importance of infusing multiple PRSs and their interactions.

## Association of AD-PRS With Cognitive Behaviors and Mental Health

The impairment of episodic memory and decline in advanced cognitive functions are the earliest and most characteristically clinical manifestations of LOAD (Bäckman et al., 2004). In the early stage, cognitive behaviors and mental health of the LOAD patients are partially impaired, which complicate and intertwine with the occurrence and progression of LOAD. Exploring the association between AD-PRS and cognitive functions has aroused many important findings. For example, AD-PRS was reported to be associated with lower total, verbal, and performance intelligence quotients in childhood and adolescence (Korologou-Linden et al., 2019a), whereas no significant associations were identified in the cognitively normal adult individuals (Li et al., 2018). Moreover, increasing studies showed that AD-PRS had a significant negative correlation with immediate memory and verbal episodic memory, which increases the predictive efficiency of conversion from healthy controls to LOAD (Marden et al., 2016; Porter et al., 2018b). It is worth noting that, in a study of Chinese samples, a significant correlation between AD-PRS and episodic memory ability was not found (Li et al., 2018). The inconsistency may be caused by ethnic differences or the evaluation efficiency of different memory scales.

AD-PRS was found to be closely associated with economic behaviors. Individuals with different levels of AD-PRS showed different saving behaviors and wealth composition (Shin et al., 2019), for instance, individuals with higher AD-PRS are more likely to hold less wealth in the Individual Retirement Accounts and to have simpler managed assets, such as fixed deposits, whereas individuals with lower AD-PRS have more complex managed assets, such as stocks (Shin et al., 2019). In addition, it was suggested that the interaction between higher AD-PRS and lower wealth levels would lead to the early-onset age of LOAD and accelerate its development (Ajnakina et al., 2020).

Mental health is also a vital risk factor affecting the onset and progression of LOAD, and up to 50% of LOAD patients have psychosis symptoms, such as hallucinations and delusions (Creese et al., 2019). Studies have shown that AD-PRS is positively correlated with neuroticism (Duberstein et al., 2011; Terracciano and Sutin, 2019) and hallucinations (Kusters et al., 2020). The association between AD-PRS and cognition was also mediated by these two personality traits (Stephan et al., 2018). Further, a combination between AD-PRS and major depression disorder-PRS has been used to study LOAD and their integration would significantly increase the ability to predict conversion from aMCI to LOAD (Xu et al., 2018). The above results indicated that LOAD shared a highly genetic association with mental health disorders.

## Opportunities and Challenges for AD-PRS Applications

AD-PRS has been widely used in many different research fields and has exhibited a huge ability in the prediction of LOAD. However, there was large heterogeneity in AD-PRS considering the huge variations in the calculation pipeline (Choi et al., 2020).

First, the selection of a certain *P*_*T*_ threshold from the GWAS summary statistics of the discovery sample was quite important for building PRS in the target sample, because it determined how many SNPs were included for calculation. In the classic AD-PRS calculation method, only those SNPs less than a predefined *P*_*T*_ threshold were included (Axelrud et al., 2018). Recently, the optimal *P*_*T*_ threshold method was applied widely, in which a series of AD-PRS were typically calculated over a range of thresholds, and the associations between the target trait and each AD-PRS were calculated to find out the best prediction model with the underlying *P*_*T*_ threshold accordingly was set as the optimized *P*_*T*_ threshold in the calculation of PRS (Choi et al., 2020). Second, after identifying the *P*_*T*_ threshold, the calculation strategies of PRS in the target sample also varied. The simple AD-PRS only calculates the number of risk alleles assuming that all SNPs have the same effect on the disease. More commonly, an odds-ratio-weighted PRS was calculated for each individual as the sum of the count of risk alleles multiplied by the corresponding effect sizes across these SNPs. Third, the quality of the base sample and target sample including ethnicity, sample size, and the number of genetic variants used has a great impact on the AD-PRS and will exert the findings. To date, no consensus has been reached about these points and various strategies have been adopted by researchers, which of course will hamper the utility of the AD-PRS for a clinical diagnosis.

Besides the above points, another important question is whether the APOE*-*ε*4* should be included for calculating AD-PRS, which is the largest risk factor for LOAD (Kim et al., 2009). At present, the accuracy of predicting the risk of LOAD by using the PRS method is 84% (Escott-Price et al., 2015, 2017). However, by far, the *APOE-*ε*4* allele (risk) and the *APOE-*ε*2* allele (protective) contributed the largest to this risk, where the predictive accuracy could reach 0.68 (*APOE-*ε*4*) and 0.69 (*APOE-*ε*4*+*APOE-*ε*2*) in the clinical samples (Escott-Price et al., 2015). An important practical and theoretical consideration is to understand how good AD-PRS is when excluding the *APOE-*ε*4* gene risk and no consensus has been reached so far. Thus, associations of the AD-PRS with multiple biomarkers adjusting for *APOE* locus or not need to be tested.

It should be noted that, although some limitations about AD-PRS still need to be addressed, the advanced development of large-GWAS studies and data-sharing policies are driving the AD-PRS to be constantly optimized and updated for drawing unambiguous conclusions about LOAD. For example, many researchers have identified that AD-PRS was associated with lower hippocampal volume in different target samples using different *P*_*T*_when using the publicly available International Genomics of Alzheimer's Project (IGAP) as the base sample (Mormino et al., 2016; Axelrud et al., 2018; Heidi et al., 2021; Tank et al., 2022). The underlying reason may be that the base sample from IGAP or UK Biobank is very large which can reduce the deviation caused by a small sample, and also offer the same risk alleles for the AD-PRS calculation which makes the most important risk alleles always included.

In the future, more studies considering the causal inference between AD-PRS, biomarkers, and LOAD occurrence are needed to infer the underlying mechanism of LOAD. Moreover, the application of AD-PRS would also be critical for drug discovery, as drugs targeting proteins encoded in genetic risk loci would be more likely to be successful in phases II and III clinical trials (King et al., 2019). Thus, AD-PRS have a greater utility in biomedical research and personalized precision medicine in the future.

## Author Contributions

QL, XL, and JX contributed to conception and design of the study. QL and XL wrote the first draft of the manuscript. JX wrote sections of the manuscript. All authors contributed to manuscript revision, read, and approved the submitted version.

## Funding

This work was supported by the National Natural Science Foundation of China (grant no. 81801687), Science & Technology Development Fund of Tianjin Education Commission for Higher Education (grant no. 2019KJ195), and Open Research Project of The Beijing Key Laboratory of High Dynamic Navigation Technology under grant no. HDN2020102.

## Conflict of Interest

The authors declare that the research was conducted in the absence of any commercial or financial relationships that could be construed as a potential conflictof interest.

## Publisher's Note

All claims expressed in this article are solely those of the authors and do not necessarily represent those of their affiliated organizations, or those of the publisher, the editors and the reviewers. Any product that may be evaluated in this article, or claim that may be made by its manufacturer, is not guaranteed or endorsed by the publisher.
